# Supplementary material for: Temporal-spatial changes and tourism efficiency coupling in Guangdong-Hong Kong-Macao Greater Bay Area
Source: PLoS One. 2025 Mar 19;20(3):e0313985. doi: 10.1371/journal.pone.0313985 (PMC11922294; doi:10.1371/journal.pone.0313985)
Supplement: Attachment — Submitted filename: comment on PONE review.docx [file pone.0313985.s001.docx]

This study investigates the temporal-spatial changes and the coupling coordination relationship of tourism efficiency and scale in the GBA. It delves into the spatial-temporal disparities in tourism efficiency and scale from economic and geographic perspectives. The manuscript offers a model of coupled and coordinated development, and puts forth corresponding recommendations for regional tourism development in the GBA. The manuscript is well organized. However, some interpretations are missing. Given this, my recommendation is Major Revision. Bellows are my specific comments:

1. In Abstract, ESDA should be given a full name when it first appears.
2. Please check the entire manuscript. For nouns that require abbreviations, use the full name and indicate the abbreviation when the abstract and introduction first appear, and hold the abbreviation status in the following text.
3. In introduction, while the role and importance of the bay area economy and tourism in the global context are well-articulated. However, when describing the tourism resources and development level of the GBA, please provide specific examples or data.
4. The scientific literature can be enriched, particularly for socioeconomical development of the GBA. The following publication can be considered.

- He, Y., Wu, L., Liang, Y., Zheng, Y., Zhang, L. (2024). Spatial suitability between water supply pressure and waterworks water supply capacity in the Guangdong-Hong Kong-Macau Greater Bay Area: Spatial-temporal evolution pattern, driving mechanism and Implications. Journal of Cleaner Production, 434, 140317.

5. Please supplement the background information and tools of ESDA while describing in detail why these methods and results are selected.
6. In literature review, please add a brief summary of the content, methodology, and results of the individual studies in the literature, and provide your opinion on the similarities, differences, and strengths of this study in relation to your research.
7. Formulas and their numbers need to be unified alignment standards, such as formula center and number right alignment.

8. Figure 1 is odd, please consider restructure it.

9. Please check the expression of the proper noun in the text and write it uniform. e.g. “Lisa cluster diagram” or “LISA cluster map”?

10. Figure 2 is odd, please consider restructure it. You can put two small maps in a row for better visualization, add place names and compasses to the maps.

11. When describing the research methodology and tools, can you give more details on the design rationale and application methods?

12. In Figure 9, add labels and compasses to the maps.

13. Please provide brief background information and definitions of terms used in the paper the first time they appear. e.g. "Coupling Coordination Degree", "Comprehensive Efficiency", etc.

14. In conclusion and discussion, please engage in a more profound analysis of the results and compare them with existing literature.

15. Please thoroughly check and revise the expression and grammar of the entire manuscript. Your manuscript needs to be carefully edited by a professional English editor, paying special attention to English grammar, spelling and sentence structure.
