# Supplementary material for: Temporal-spatial changes and tourism efficiency coupling in Guangdong-Hong Kong-Macao Greater Bay Area
Source: PLoS One. 2025 Mar 19;20(3):e0313985. doi: 10.1371/journal.pone.0313985 (PMC11922294; doi:10.1371/journal.pone.0313985)
Supplement: Attachment — Submitted filename: Response to Reviewers1.doc [file pone.0313985.s003.doc]

Response to Reviewers’ comment

Response to Reviewers’ comment

Manuscript No. PONE-D-24-04406

Title:Study on Temporal-spatial Changes and Tourism Efficiency Coupling in Guangdong-Hong Kong-Macao Greater Bay Area

**Responses to reviewer 1:**

First, we would like to thank you for your positive feedback of the paper and providing us very useful and constructive comments and suggestions. Listed below are the point-by-point responses to the reviews’ comments. We hope our revision may improve the paper. In the new version of our paper, the modified parts have been highlighted in red.

**Comment 1**: In Abstract, ESDA should be given a full name when it first appears.

Response: ESDA has be given a full name as “Earth Science Data Acquisition”.

**Comment 2**: Please check the entire manuscript. For nouns that require abbreviations, use the full name and indicate the abbreviation when the abstract and introduction first appear, and hold the abbreviation status in the following text.

Response: We have made modifications to the entire manuscript according to your suggestions.

**Comment 3:** In introduction, while the role and importance of the bay area economy and tourism in the global context are well-articulated. However, when describing the tourism resources and development level of the GBA, please provide specific examples or data.

Response: Increased data on inbound tourism and high-level tourism resources in GBA.

**Comment 4:** The scientific literature can be enriched, particularly for socioeconomical development of the GBA. The following publication can be considered.

Response: According to your suggestion, some literature related to GBA has been added, including the one you suggested.

**Comment 5:** Please supplement the background information and tools of ESDA while describing in detail why these methods and results are selected.

Response: 3.1.4 provides a detailed introduction to ESDA and an analysis of the reasons for choosing this method.

**Comment 6:** In literature review, please add a brief summary of the content, methodology, and results of the individual studies in the literature, and provide your opinion on the similarities, differences, and strengths of this study in relation to your research.

Response: The first two paragraphs of “3.Methodology” in Page6 provide a brief review of the literature and their reference and inspiration for this study.

**Comment 7:** Formulas and their numbers need to be unified alignment standards, such as formula center and number right alignment.

Response: We have made adjustments and standardized according to your suggestions.

**Comment 8:** Figure 1 is odd, please consider restructure it.

Response: We have made adjustments to Figure 1 according to your suggestion.

**Comment 9:** Please check the expression of the proper noun in the text and write it uniform. e.g. “Lisa cluster diagram” or “LISA cluster map”?

Response: We have rechecked the expression of specific nouns in the text according to your suggestion and made unified writing.

**Comment 10:** Figure 2 is odd, please consider restructure it. You can put two small maps in a row for better visualization, add place names and compasses to the maps.

Response: We have made adjustments according to your suggestion and added place names and compasses to the maps.

**Comment 11:** When describing the research methodology and tools, can you give more details on the design rationale and application methods?

Response:

(1)DEA-BCC model: Further detailed explanations were provided for the DEA-BCC model in 3.1.1.

(2)Malmquist productivity index model:Detailed introduction has been provided in 3.1.2.

(3)Exploratory spatial data analysis(ESDA):Detailed introduction has been provided in 3.1.3.

**Comment 12:** In Figure 9, add labels and compasses to the maps.

Response: We have made adjustments according to your suggestion and added place names and compasses to the maps.

**Comment 13:** Please provide brief background information and definitions of terms used in the paper the first time they appear. e.g. "Coupling Coordination Degree", "Comprehensive Efficiency", etc

Response: Part of the paper briefly introduces the background and definitions, such as:

Coupling and coordination model:

Coupling degree is an index to judge the degree of interaction between two or more elemental systems based on quantitative data (Wang Cheng, Tang Ning, 2018).(page9)

Coupling Coordination Degree is an index to quantitatively judge the goodness of coupling between two or more element systems, and it is a further analysis of the coupling state of tourism efficiency and scale.

**Comment 14:** In conclusion and discussion, please engage in a more profound analysis of the results and compare them with existing literature.

Response: Supplemented literature in similar Chinese contexts.

**Comment 15:** Please thoroughly check and revise the expression and grammar of the entire manuscript. Your manuscript needs to be carefully edited by a professional English editor, paying special attention to English grammar, spelling and sentence structure.

Response: A professional English editor has been invited to re edit the paper.

**Responses to reviewer 2:**

First, we would like to thank you for your positive feedback of the paper and providing us very useful and constructive comments and suggestions. Listed below are the point-by-point responses to the reviews’ comments. We hope our revision may improve the paper. In the new version of our paper, the modified parts have been highlighted in red.

**Comment 1:** Can you provide more details on the specific data sources used for analyzing tourism efficiency and scale in the Guangdong-Hong Kong-Macao Greater Bay Area? How were these data collected and processed to ensure accuracy and reliability in the analysis?

Response:The specific data sources are detailed in 3.2.2, and the selection of indicators is detailed in 3.2.1.

**Comment 2:** Could you elaborate on the methodology employed to measure tourism efficiency using the DEA-BCC model and the total factor productivity index model? How were these models applied in the context of regional tourism development, and what were the key considerations in their implementation?

Response: Further detailed explanations were provided for the DEA-BCC model in 3.1.1.

The total factor productivity index model is calculated using the DEA-Malmquist productivity index model. The detailed calculation process of the DEA Malmquist productivity index model is described in 3.1.2.

**Comment 3:** In the exploration of temporal and spatial differentiation of tourism efficiency and scale, what were the main findings regarding the evolution of these factors over the study period from 2001 to 2019? How did the coupling analysis reveal insights into the relationship between tourism efficiency and scale in the region?

Response:The main findings of this study were analyzed in detail in section 4. Results from 11 to 25. The spatiotemporal differences in tourism efficiency and scale in GBA are mainly analyzed from the static characteristics of tourism efficiency and scale, the dynamic characteristics of tourism efficiency, the dynamic characteristics of tourism scale, the spatiotemporal dynamic changes of tourism efficiency, and the spatiotemporal dynamic changes of tourism scale. The coupling and coordination relationship between tourism efficiency and tourism scale in GBA is mainly analyzed from the aspects of coupling degree spatiotemporal differentiation and coupling coordination degree spatiotemporal differentiation.

A brief summary of the results is presented in 5.1 Conclusions.

**Comment 4:** Can you discuss the implications of the study's results on the collaborative development and marketing strategies for enhancing the quality of travel services in the Greater Bay Area? How do the findings contribute to the understanding of regional tourism economies and their impact on overall economic development?

Response:Your proposal is of great help to the subsequent research on tourism marketing in the GBA, but the theme of this article is to explore the spatiotemporal changes and coordinated development mechanisms of 11 cities in GBA through historical data.

As for how to help understand the regional tourism economies and their impact on overall economic development, it is stated in the Practical implications.

**Comment 5:** Have you considered conducting sensitivity analyses or robustness checks to validate the stability and reliability of the results obtained from the DEA-BCC model and the total factor productivity index model? How do you address potential uncertainties or variations in the data that may affect the conclusions drawn from the study?

Response:The robustness test of panel data is supplemented in section 3.2.3.
