# Supplementary material for: Temporal-spatial changes and tourism efficiency coupling in Guangdong-Hong Kong-Macao Greater Bay Area
Source: PLoS One. 2025 Mar 19;20(3):e0313985. doi: 10.1371/journal.pone.0313985 (PMC11922294; doi:10.1371/journal.pone.0313985)
Supplement: Attachment — Submitted filename: renamed_8497e.doc [file pone.0313985.s004.doc]

Response to Reviewers’ comment

Manuscript No. PONE-D-24-04406

Title:Study on Temporal-spatial Changes and Tourism Efficiency Coupling in Guangdong-Hong Kong-Macao Greater Bay Area

Dear Editor,

We appreciate you very much for your positive and constructive comments on our manuscript. We have fully revised our manuscript and have addressed all of reviewer’s comments. The detailed revisions are listed below and highlighted in the revised manuscript with red background. We hope that the revisions in the resubmitted manuscript and our responses will be sufficient to make our manuscript meets your high standards.

Thank you for your consideration and time.

*Sincerely yours,*

*Kaijun Wu**

*International Cruise & Yacht College of Guangzhou Maritime University, Guangzhou 510725, China, gzwkj@126.com*

*Xilin Yang,*

*School of Psychology, Shenzhen University, Shenzhen 518000,China, 1711945825@qq.com*

*Xingfu Han*

*Culture Tourism & Geography School of Guangdong University of Finance & Economics, Guangzhou 510320, China, 2354780221@qq.com*

Response to Editor’ comment

Manuscript No. PONE-D-24-04406

Title:Study on Temporal-spatial Changes and Tourism Efficiency Coupling in Guangdong-Hong Kong-Macao Greater Bay Area

**Responses to editor:**

First, we would like to thank you for your positive feedback of the paper and providing us very useful and constructive comments and suggestions again. Listed below are the point-by-point responses to editor’ comments. We hope our second revision may improve the paper. In the new version of our paper, the modified parts have been highlighted in blue.

**Comment 1**: About the data available on acceptance.

Response: We have provided a data sharing link.

link: https://pan.baidu.com/s/14SaDRfgjn3mTyZSWsMgooQ	Extracted code: 2azu

**Comment 2**: About figure 2, 6, 8 and 9 in your submission.

Response: Because we were unable to obtain permission, we changed the corresponding figures to be presented in the form of tables. Correspondingly, convert Figure 2 into Table 2, Figure 6 into Table 4, Figure 8 into Table 5, and Figure 9 into Table 6.

Response to Reviewers’ comment

Manuscript No. PONE-D-24-04406

Title:Study on Temporal-spatial Changes and Tourism Efficiency Coupling in Guangdong-Hong Kong-Macao Greater Bay Area

**Responses to reviewer 1:**

First, we would like to thank you for your positive feedback of the paper and providing us very useful and constructive comments and suggestions again. Listed below are the point-by-point responses to the reviews’ comments. We hope our second revision may improve the paper. In the new version of our paper, the modified parts have been highlighted in red.

**Comment 1**: The authors made some improvements, however, references of the revised version should be appropriately checked and improved.

Response: We have carefully checked and revised the references.

**Responses to reviewer 3:**

First, we would like to thank you for your positive feedback of the paper and providing us very useful and constructive comments and suggestions again. Listed below are the point-by-point responses to the reviews’ comments. We hope our second revision may improve the paper. In the new version of our paper, the modified parts have been highlighted in green.

**Comment 1:** Some of the words used are inconsistent, such as the introduction part “the coupled and coordinated development model” is inconsistent with similar expressions in the abstract.

Response: We have carefully examined the inconsistent expressions in the text and have unified them.

**Comment 2:** The research methodology is relatively common, so it is not necessary to introduce it at length.

Response: During the revision of the first draft, Reviewer 1 suggested that some research methods require more detailed explanations, so additional modifications were made based on the original manuscript.

**Comment 3:** The results of the empirical analyses lack a brief analysis of the causes.

Response: The results of the empirical analysis actually include a brief analysis of the causes, such as the possible reasons for the results of some cities in 4.1.1. 1) in green font.

**Comment 4:** The authors should double-check the coupling formula.

Response: We have carefully checked the coupling degree formula and coupling coordination degree model again, and there should be no problem. If there are any further errors, please point them out and we will make the necessary revisions.

**Comment 5:** On what basis is the grading of coupling coordination degree classified? Please give the basis.

Response:We have added the grading criteria for coupling coordination degree in the last paragraph of 3.1.5.

**Comment 6:** The images layout must be further adjusted, for example, the size and position of Fig. 9(b) fails to be consistent with several other figures, please make reasonable adjustments.

Response: We make reasonable adjustments to the layout such as image size and position.

**Comment 7:** Indicators are cited with reasons, but the reference articles are not clearly indicated.

Response: We have added reference annotations for the indicators.

**Comment 8:** The expression of the conclusion is not concise enough, and some of the contents of the conclusion can be incorporated into the discussion.

Response:Based on your suggestion, we have simplified the expression of the conclusion.

**Comment 9:** “Theoretical impact” in the discussion is to express the similarities and differences between this article and related studies?

Response:There is no 'Theoretical impact' in the manuscript, only 'Theoretical implications'. We further refined the theoretical and practical implications.

**Comment 10:** The innovative points or marginal contributions of the article need to be more clearly reflected.

Response:We further refined the theoretical and practical implications.
