# Supplementary material for: Temporal-spatial changes and tourism efficiency coupling in Guangdong-Hong Kong-Macao Greater Bay Area
Source: PLoS One. 2025 Mar 19;20(3):e0313985. doi: 10.1371/journal.pone.0313985 (PMC11922294; doi:10.1371/journal.pone.0313985)
Supplement: Attachment — Submitted filename: renamed_429b2.doc [file pone.0313985.s005.doc]

Response to Reviewers’ comment

Manuscript No. PONE-D-24-04406

Title:Study on Temporal-spatial Changes and Tourism Efficiency Coupling in Guangdong-Hong Kong-Macao Greater Bay Area

Dear Editor,

We appreciate you very much for your positive and constructive comments on our manuscript. We have fully revised our manuscript and have addressed all of reviewer’s comments. The detailed revisions are listed below and highlighted in the revised manuscript with red background. We hope that the revisions in the resubmitted manuscript and our responses will be sufficient to make our manuscript meets your high standards.

Thank you for your consideration and time.

*Sincerely yours,*

*Kaijun Wu**

*International Cruise & Yacht College, Guangzhou Maritime University, Guangzhou 510725, China, gzwkj@126.com*

*Binlan Wu**

*School of Economics and Management, South China Normal University, Guangzhou 510000, China, binglanwu@foxmail.com*

*Xilin Yang,*

*School of Psychology, Shenzhen University, Shenzhen 518000, China, 1711945825@qq.com*

*Wanfu Jin*

*School of Geography and Environmental Economics, Guangdong University of Finance & Economics, Guangzhou 510320, China, jinwanfu0927@163.com*

Response to Reviewers’ comment

Manuscript No. PONE-D-24-04406

Title:Study on Temporal-spatial Changes and Tourism Efficiency Coupling in Guangdong-Hong Kong-Macao Greater Bay Area

**Responses to editor:**

First, we would like to thank you for your positive feedback of the paper and providing us very useful and constructive comments and suggestions again. Listed below are the point-by-point responses to the editor’ comments. We hope our fourth revision may improve the paper.

**Comment 1**: Drop "study on" from the title.

**Response:** We have dropped them.

**Comment 2**: The author excessively self-cites, and irrelevant self-citations need to be removed.

**Response:** We have removed references 47, 48, 49.

**Comment 3**: A thorough revision is required.

**Response:** We have made a one-step revision and improvement to the paper.

**Comment 4**: The map creation in the paper is not up to standard; it is recommended that the author seek review from a scholar with a background in cartography.

**Response:** We asked scholars with a background in geography to remake the map and clearly indicate the source of the original map in **Acknowledgments**.

**Comment 5**: The academic rigor of the paper needs to be improved.

**Response:** We have further improved the academic rigor of the paper.

**Responses to reviewer 1:**

First, we would like to thank you for your positive feedback of the paper and providing us very useful and constructive comments and suggestions again. Listed below are the point-by-point responses to the reviews’ comments. We hope our fourth revision may improve the paper. In the new version of our paper, the modified parts have been highlighted in red.

**Comment 1**: The authors made some improvements, however, references of the revised version should be appropriately checked and improved.

Response: We have carefully checked and revised the references.

**Responses to reviewer 3:**

First, we would like to thank you for your positive feedback of the paper and providing us very useful and constructive comments and suggestions again. Listed below are the point-by-point responses to the reviews’ comments. We hope our fourth revision may improve the paper. In the new version of our paper, the modified parts have been highlighted in green.

**Comment 1:** Some of the words used are inconsistent, such as the introduction part “the coupled and coordinated development model” is inconsistent with similar expressions in the abstract.

Response: We have carefully examined the inconsistent expressions in the text and have unified them.

**Comment 2:** The research methodology is relatively common, so it is not necessary to introduce it at length.

Response: During the revision of the first draft, Reviewer 1 suggested that some research methods require more detailed explanations, so additional modifications were made based on the original manuscript.

**Comment 3:** The results of the empirical analyses lack a brief analysis of the causes.

Response: The results of the empirical analysis actually include a brief analysis of the causes, such as the possible reasons for the results of some cities in 4.1.1. 1) in green font.

**Comment 4:** The authors should double-check the coupling formula.

Response: We have carefully checked the coupling degree formula and coupling coordination degree model again, and there should be no problem. If there are any further errors, please point them out and we will make the necessary revisions.

**Comment 5:** On what basis is the grading of coupling coordination degree classified? Please give the basis.

Response:We have added the grading criteria for coupling coordination degree in the last paragraph of 3.1.5.

**Comment 6:** The images layout must be further adjusted, for example, the size and position of Fig. 9(b) fails to be consistent with several other figures, please make reasonable adjustments.

Response: We make reasonable adjustments to the layout such as image size and position.

**Comment 7:** Indicators are cited with reasons, but the reference articles are not clearly indicated.

Response: We have added reference annotations for the indicators.

**Comment 8:** The expression of the conclusion is not concise enough, and some of the contents of the conclusion can be incorporated into the discussion.

Response:Based on your suggestion, we have simplified the expression of the conclusion.

**Comment 9:** “Theoretical impact” in the discussion is to express the similarities and differences between this article and related studies?

Response:There is no 'Theoretical impact' in the manuscript, only 'Theoretical implications'. We further refined the theoretical and practical implications.

**Comment 10:** The innovative points or marginal contributions of the article need to be more clearly reflected.

Response:We further refined the theoretical and practical implications.
