# Supplementary material for: Temporal-spatial changes and tourism efficiency coupling in Guangdong-Hong Kong-Macao Greater Bay Area
Source: PLoS One. 2025 Mar 19;20(3):e0313985. doi: 10.1371/journal.pone.0313985 (PMC11922294; doi:10.1371/journal.pone.0313985)
Supplement: Attachment — Submitted filename: renamed_c6050.doc [file pone.0313985.s006.doc]

Response to Reviewers’ comment

Manuscript No. PONE-D-24-04406

Title:Temporal-spatial Changes and Tourism Efficiency Coupling in Guangdong-Hong Kong-Macao Greater Bay Area

Dear Editor,

We appreciate you very much for your positive and constructive comments on our manuscript. We have fully revised our manuscript and have addressed all of your comments. The detailed revisions are listed below and highlighted in the revised manuscript with a red background. We hope that the revisions in the resubmitted manuscript and our responses will be sufficient to make our manuscript meet your high standards.

Thank you for your consideration and time.

*Sincerely yours,*

*Kaijun Wu**

*School of Maritime Law and Traffic Management, Guangzhou Maritime University, Guangzhou 510725, China, gzwkj@126.com*

*Xilin Yang,*

*School of Psychology, Shenzhen University, Shenzhen 518000, China, 1711945825@qq.com*

*Xingfu Han*

*Huawei Institute of Information, Communication and Technology，Guangdong Baiyun University, Guangzhou 510450, China, 2354780221@qq.com*

Response to Reviewers’ comment

Manuscript No. PONE-D-24-04406

Title: Temporal-spatial Changes and Tourism Efficiency Coupling in Guangdong-Hong Kong-Macao Greater Bay Area

**Responses to editor:**

First, we would like to thank you for your positive feedback on the paper and for providing us very useful and constructive comments and suggestions again. Listed below are the point-by-point responses to the editor’s comments. We hope our eighth revision may improve the paper.

**Comment 1**: About the problem of the underlying data set.

**Response:** The source of the underlying data set can be found in section 3.2.2 of the manuscript and there is a detailed explanation in Data Sources. The specific links to the underlying data required for the relevant research are as follows:

(1) http://stats.gd.gov.cn/gdtjnj/index.html
(2) https://gddata.gd.gov.cn/opdata/index?chooseValue=collectForm&deptCode=48
(3) https://www.censtatd.gov.hk/sc/EIndexbySubject.html?pcode=B1010003&scode=420
(4) https://www.dsec.gov.mo/zh-CN/
(5) https://partnernet.hktb.com/china/sc/home/index.html

The specific links to the underlying summary data and data analysis results are as follows: https://pan.baidu.com/s/1rDvTBB0xSrrtjxyK_UkEaw?pwd=c95w Extracted code:c95w.
